# Mortality in Edentulous Patients: A Registry-Based Cohort Study in Sweden Comparing 8463 Patients Treated with Removable Dentures or Implant-Supported Dental Prostheses

**DOI:** 10.1155/2021/9919732

**Published:** 2021-07-30

**Authors:** Jan Kowar, Victoria Stenport, Mats Nilsson, Torsten Jemt

**Affiliations:** ^1^Brånemark Clinic, Public Dental Health Care Service, Region of Västra Götaland, Gothenburg, Sweden; ^2^Department of Prosthetic Dentistry/Dental Material Science, The Sahlgrenska Academy at Göteborg University, Gothenburg, Sweden; ^3^Futurum, Academy of Health and Care, Region Jönköping County, Jönköping and Department of Medical and Health Sciences, Linköping University, Linköping, Sweden

## Abstract

**Objectives:**

The purpose of this study was to investigate if edentulism is associated with all-cause mortality. The aims were to analyze the association between age, socioeconomic factors, and mortality in edentulous patients treated with either removable dentures or implant-supported prostheses.

**Methods:**

All patients who became edentulous according to the Swedish Social Insurance Agency (SSIA) between 2009 and 2013 (*N*  = 8463) were analyzed regarding prosthetic treatment, age, gender, and socioeconomic status. The patients were divided into two groups, depending on whether they were treated with dental implants (implant group; IG) or with conventional removable dentures (denture group; DG). Data on mortality for all included individuals were obtained from the Swedish National Cause of Death Register and compared to a reference population. Cumulative survival rates were calculated, and a multivariable regression analysis for the included variables was performed.

**Results:**

Between 2009 and 2018, 2192 of the patients (25.9%) were treated with implant-supported dental prostheses (IG) and 6271 patients (74.1%) were treated with removable dentures without support of dental implants (DG). Altogether 2526 patients (30%) died until December 31, 2019, and the overall mortality was significantly higher for the DG compared to the IG during follow-up (*p* < 0.001). Younger edentulous patients (≤59 years) presented a higher mortality than the reference population, while implant patients over 79 years of age demonstrated a lower mortality. The final results from the multivariable logistic analysis showed that lower equalized disposable income (EDI) and the choice of conventional removable dentures are the most important factors for increased patient mortality (*p* < 0.001).

**Conclusions:**

Edentulous patients have an overall higher mortality compared to a reference population. Low socioeconomic status increases all-cause mortality. Individuals treated with dental implants show statistically significant lower 10-year mortality compared to patients treated with conventional removable dentures, regardless of socioeconomic status.

## 1. Introduction

Oral health is one of the topics that the World Health Organization (WHO) has on its agenda. The WHO declares that oral health is a key indicator of the overall health, well-being, and quality of life (QoL). The oral health is affected by a wide range of oral diseases such as dental caries, periodontal disease, oral cancer, orodental trauma, and also general diseases as diabetes, immunomodulating diagnoses, and medications among others. The Global Burden of Disease Study 2017 estimated that oral diseases affect nearly 3.5 billion people worldwide, with untreated dental caries in permanent teeth being the most common condition [1]. Orodental trauma is the 5^th^ most prevalent disease/injury in the world [2] and the treatment is often difficult and costly and can sometimes even lead to tooth loss, resulting in complications for facial and psychological development and decreased QoL [3]. Severe periodontal disease is another widespread oral disorder which may result in tooth loss and affects almost 10% of the global population [1]. Dental caries, severe periodontitis, trauma, and cancer are the most common reasons for severe tooth loss and edentulism [4–6].

The prevalence of edentulism in the adult (>15 years of age) worldwide population was estimated at 4.8% (267.5 million) in 2017 and about 18.5 million people became edentulous this year [1]. In Sweden, the prevalence of edentulous individuals decreased dramatically over the last decades and nowadays it is estimated that 2.7% of the 65- to 74-year-old individuals are edentulous [7] with prevalence as low as 0.3% in the age group 40–70 years of age [8]. It is considered important to rehabilitate edentulous patients to ensure masticatory function as well as esthetical appearance aesthetic for social well-being. There is evidence of a relationship between malnutrition and edentulousness in older individuals [9] and unfavorable socioeconomic conditions have been associated with higher numbers of edentulous individuals [10]. The adequate dental treatment of edentulism can simply be divided into rehabilitation with conventional removable complete dentures and rehabilitation with implant-supported prostheses. Implant-supported prostheses have become more and more common over the last decades after the invention of the osseointegrated dental implants by Brånemark in the 1960s [11]. Implant-supported prostheses are today considered as an established treatment option in the edentulous jaw. However, the treatment is time-consuming and costly compared to the rehabilitation with conventional removable dentures.

In addition to functional and aesthetic aspects, other observations associated with edentulism have been discussed over the last years. The number of remaining teeth has shown to be a predictor for all-cause mortality [12] as well as circulatory mortality [13]. The mortality rate measures the mortality from all causes or cause-specific diseases in a given time interval in a population and is an incidence rate where the event being measured is death. Some studies report that younger age groups of edentulous patients have higher mortality rates compared to a reference population adjusted for age, gender, and year of treatment [14, 15] while others support the opposite [16].

Mortality in association with different age groups has been discussed previously in dentistry [14] and an association between prosthetic treatment, wearing complete dentures, and mortality has been suggested [17, 18], indicating that denture wearing may be associated with a decreased all-cause mortality in edentulous conditions. However, a causal relationship between denture wearing and decreased mortality in edentulous patients has not yet been identified.

Association between edentulism and mortality could be affected by socioeconomic status (SES). However, these factors are often difficult to define, and the variables used to explain SES are not easy to interpret [16]. In a Swedish population study [19], obesity was associated with edentulism, most obvious in women aged 55–74 years. Mack et al. [20] found that low education level is associated with higher risk for edentulism and in a recent published cohort study [21] from Brazil the authors concluded that edentulism is a significant predictor for all-cause mortality, also after adjustment for SES factors. In-depth analysis of the correlation between edentulism, mortality, and possible influence of SES factors could be of interest.

The purpose of this study was to investigate how edentulism is associated with mortality in a Swedish population. The hypothesis was that younger age groups and low socioeconomic status increase the risk of early mortality in edentulous patients. The specific aims of the study were to analyze the association between age at the time of complete tooth loss and all-cause mortality in edentulous patients as a group and treated with either removable dentures or implant-supported prostheses.

## 2. Materials and Methods

### 2.1. Study Design

In Sweden, all dental health care providers are required to report dental care to the Swedish Social Insurance Agency (SSIA) which handles the administration of the dental care subsidy.

This retrospective closed cohort study includes all adult patients (>19 years old) who were eligible for reimbursement from the SSIA and who became edentulous in both jaws between 2009 and 2013 in Sweden. All these patients who were restored with conventional removable dentures or implant-supported fixed or removable prostheses until December 2018 were included and analyzed. The patients were divided into two groups with regard to whether they were treated with implants or not: without dental implants (denture group; DG) and with dental implants in one or both jaws (implant group; IG).

### 2.2. Patients

The data register of the SSIA was searched for individual data on edentulous patients who could be included in one of the two groups. The observation time for complete edentulousness was between January 2009 and December 2013, while the search for patients who have received a complete denture and/or an implant-supported prosthesis was tested against reported dental SSIA data until 31 December 2018. “Completely edentulous” is defined at inclusion (2009–2013) as the reported data in the register of the SSIA shows that the patient is edentulous in both jaws according to specific codes at the same time of (last) tooth extraction.

The register of the SSIA includes codes for dental treatment, either codes for conventional complete removable dentures or codes for dental implants. If one of the implant codes was registered in any jaw after inclusion of the patient and until 31 December 2018, the patient was placed in the implant group (IG). The remaining patients identified as edentulous were treated with conventional removable dentures (DG), and no untreated patient was observed after last tooth extraction.

Thus, at the time of complete denture/implant treatment at least one extraction of a natural tooth must have been reported to the SSIA register for any dental position to include the patient in the study group of becoming completely edentulous. This is to avoid including patients who have been edentulous for a long time and/or who replace an earlier complete denture with a new denture, or an implant-supported prosthesis.

### 2.3. Variables

Data on mortality for all included patients were obtained from the Swedish National Cause of Death Register until December 2019. This allows for a minimum of one-year follow-up after final dental treatment. The observed number of deceased patients was used to calculate the cumulative survival rate (CSR) for the IG and DG and compare both groups with a reference population in Sweden, adjusted for age and gender [22]. Data for remaining life expectancy at the time when the patient becomes edentulous was collected from lifetables on the Swedish population [23]. Furthermore, the observed number of deceased patients in both study groups was compared with an expected number in a reference population based on age, gender, and the calendar year-specific mortality rate from the Swedish National Cause of Death Register.

Socioeconomic and demographic data for all included patients were obtained from Statistics Sweden [24]. From the Register of Education of the Swedish Population, the highest level of education for each individual was collected and coded according to the Classification of Swedish education (SUN 2000 [25]). The latter is adapted to the International Standard Classification of Education (ISCED 97 [26]).

The Total Population Register from Statistics Sweden was searched for each included patient for the country of birth and the place of living on the municipal level. The patients were divided into two subgroups depending on if they were born in Sweden or abroad. The classification of the regional typology is applied to the Nomenclature of Territorial Units for Statistics (NUTS [27]) and identifies three types of municipalities: (1) patients living in municipalities with less than 20% (predominantly urban region), (2) up to 50% (intermediate region), or (3) more than 50% (predominantly rural region) of their population in rural regions, respectively [28].

Furthermore, information about equalized disposable income (EDI) was collected from Statistics Sweden for all included patients [24]. The EDI is the total income of a household per year, after tax and other deductions, divided by the number of household members converted into equalized adults and makes it possible to compare income between individuals in different family situations. Income levels were defined for three different groups; the limit for the lowest annual income (133 867 SEK) was defined as an income that is 50 percent lower than the median income of the whole study group while the limit for the highest annual income (307 518 SEK) was 50 percent higher than the median income. The intermediate group was defined between these two levels.

### 2.4. Statistics

Descriptive statistics for the patients are presented as numbers and percentages as well as min and max values. To analyze patient survival time from date of inclusion to either death or last day of follow-up (December 31, 2019), the Kaplan–Meier (KM) estimator has been performed to evaluate if survival, stratified on age and treatment, differs among patients. If no data were censored, the KM estimator $\hat{s}\left(t\right)=$ the proportion observations in the population with an event time greater than *t*. In this study, all the observations are right censored at the same time (December 31, 2019), and the observed event time is before December 31, 2019. This means that the KM estimator in this case also is as described above.

**Figure 1:**
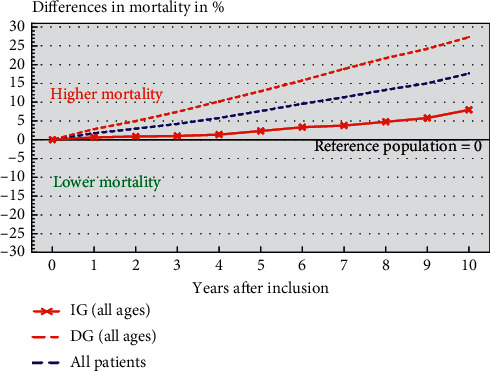
Life table survival curves for all included patients (*N*  = 8463) and for the two subgroups (DG and IG) compared to the reference population at the same age interval during 10 years of follow-up. Difference in mortality compared to the corresponding reference population was significant for all groups (*p* < 0.05).

**Figure 2:**
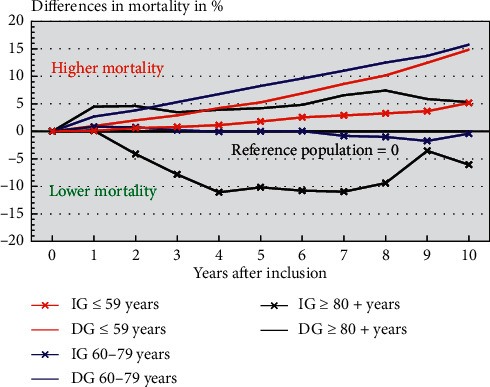
The difference in 10-year mortality between reference populations and patients treated with (IG, implant group) or without implants (DG, denture group) in different age groups over time. Difference in mortality compared to the corresponding reference population was significant for all groups (*p* < 0.05).

Survival analysis was also used to evaluate survival among patients and the reference population. As a reference population, the Swedish population over the period in the studied age groups was used. The difference in survival between patients and reference population (in percent) was calculated (Figures 1 and 2) from the difference in survival over the follow-up time in respective age group. The level for the reference population was set as zero.

To evaluate the connection between mortality and socioeconomic factors, the data were analyzed with uni- and multivariable logistic regression. The univariable logistic regression was first used to evaluate which variables that were to be included in the multivariable model. All explanatory variables with a *p* value ≤0.2 were included in the first multivariable analysis, and those variables that had a *p* value ≤0.05 were included in the semifinal model. If some of the variables in the semifinal model had a *p* value>0.05, they were excluded in the final run of the model.

In the uni- and multivariable logistic regression analysis, event (deceased, coded = 1) versus the nonevent (alive, coded = 0) were used as outcome. A logit model was used to estimate the probability of being deceased as the probability to decease before the end of the study only can take a value between 0 and 1.

SAS^®^ Stat version 13.1 software, Proc Logistic (Copyright^©^ 2002–2012 by SAS Institute Inc., Cary, NC, USA), was were used for the Kaplan–Meier and logistic regression data analysis. IBM SPSS Statistics for Windows, Version 25.0. Armonk, NY: IBM Corp IBM were used for some of the descriptive analysis.

### 2.5. Ethical Protection

The STROBE guidelines [29] for reporting observational studies were followed in the study design. This register-based study was approved by the Swedish Ethical Review Authority (Dnr 2019–02118) and a secrecy examination was conducted by the SSIA and Statistics Sweden.

## 3. Results

### 3.1. Patients and Variables

Between January 2009 and December 2013, it was possible to identify 8463 patients who became completely edentulous in the SSIA register. Thus, the overall mean annual incidence of edentulism in the present study group was 23 patients per 100,000 (0.023%) adult persons. During follow-up (2009–2018), a subgroup of 2192 patients (25.9%) were treated with implant-supported dental prostheses in at least one jaw (IG) and the remaining 6271 patients (74.1%) were treated with removable dentures without support of dental implants (DG).

**Figure 3:**
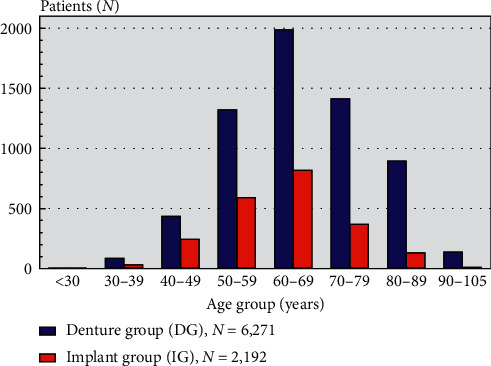
Distribution of 8463 edentulous patients with regard to age in both study groups (denture group, DG/implant group, IG).

The distribution of patients in different age groups is provided in Figure 3. The median age for the total study group was 65 years (57–74) with a range from 27 to 100 years. Altogether, 3780 of the included patients were women (45%). The median age for the IG (896 women/1296 men) and the DG group (2884 women/3387 men) was 62 years (55–69) and 66 years (58–75), respectively.

**Figure 4:**
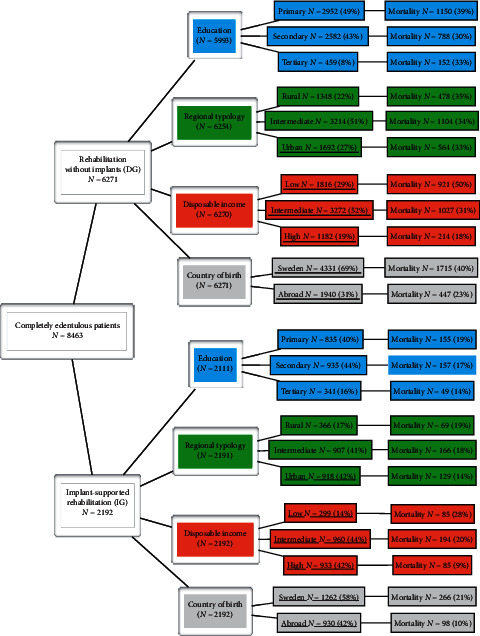
Flowchart of included edentulous patients.

The distribution of the different variables for the included patients is presented in Figure 4. Data for 359 included patients (4.4%) was missing in the Register of Education of the Swedish Population. The remaining 8104 patients (96%) are presented with regard to three subgroups according to completed education until December 2018: primary, secondary, and tertiary education level. Data for the regional typology was missing for 18 patients (0.2%) in the Total Population Register. Notably, significantly more patients in the implant group lived in urban areas and more denture patients in rural areas. The equivalized disposable income (EDI) was obtained for all included patients from Statistics Sweden, except one missing data, and divided into low-, intermediate-, and high-level income. Patients in the implant group (IG) showed higher income than those in the denture group. It was possible to track the country of birth for all included patients. Two-thirds were born in Sweden (*N*  = 5593) and the remaining patients were born abroad (*N*  = 2870).

### 3.2. Patient Mortality

Altogether, 2526 patients (30%) died until December 31, 2019. The overall mortality was significantly higher for the DG compared to the IG during follow-up (*p* < 0.001). On the other side, both groups of edentulous patients show a significantly higher overall mortality than the reference population over a ten-year follow-up period after inclusion (Figure 1).

An increased mortality compared to the reference population was observed for both groups (IG and DG) in edentulous patients younger than 60 years old (*p* < 0.001). Moreover, there was a consistent higher mortality (*p* < 0.001) for the DG compared to the IG over ten years of follow-up in all age groups (Figure 2). Patients older than 60 years, treated with dental implants (IG), show significantly lower mortality than the reference population and the differences increase with increased age (*p* < 0.05). It can be noticed that there is an increasing difference for the first four years of follow-up for patients older than 79 years of age followed by a reduced difference up to the termination of the observation period in the implant group (Figure 2).

As shown in Figure 4, the mortality rate is generally higher for all included variables in patients treated with removable dentures (DG) compared to those in the IG. When comparing the two study groups (IG and DG) in a univariable logistic analysis; age at inclusion, the EDI (income), and the country of birth show the highest association to mortality in both study groups. Female patients and those participants from the implant group who lived in urban areas showed statistically significantly lower mortality (OR 0.8 and OR 0.7; *p* < 0.05). No statistically significant differences with respect to education level and for the regional typology for patients treated with implants were obtained after the univariable analyses and these variables were excluded in the final multivariable analyses (*p* > 0.05).

The results from the multivariable logistic analysis within the two study groups (Table 1) show that all included variables, except education level (for both groups) and regional typology (only for the IG), contributed to a statistically significant difference in mortality in the final analysis. Higher age at the time of inclusion and lower EDI are the two most important factors for expected patient mortality in the groups (Table 1). However, when comparing between the groups, no statistically significant difference for the two variables was observed (*p* > 0.05).

The results of the multivariable analysis within the groups stratified for age confirm that low income (EDI) is associated with higher risk of mortality (*p* < 0.05). Also, if the patient was treated with dental implants, the risk of mortality was lower in all age groups (Table 2; *p* < 0.05). Male patients presented a higher mortality risk compared to women regardless of age at the time of inclusion (Table 2; *p* < 0.05) and type of dental treatment (Table 1; *p* < 0.05). It can also be noted that individuals born abroad have a lower mortality risk, especially in the youngest (OR = 1.9) age group (Table 2; *p* < 0.05).

## 4. Discussion

### 4.1. Key Results

The findings of the present study showed that completely edentulous individuals in Sweden had a 18% increased 10-year mortality risk compared to a reference population. This is in agreement with previous publications [30–33] where edentulous patients presented higher mortality patterns as compared to the reference populations. Paganini Hill et al. [31] reported that edentulous men and women in the US had a 30% mortality increase compared to patients with 20 or more remaining teeth. In two different studies from Scandinavia, edentulous individuals had a 2.8 higher mortality risk over a period of seven [32] and ten years of follow-up [33], respectively.

The present study includes 8463 patients who became edentulous over a five-year period of time in Sweden, which corresponds to an estimated annual incidence of about 0.023% of the adult population. This suggests that edentulousness nowadays is a rare disease in Sweden. The highest proportion of included patients was observed at an age of 65 years with a decreasing number for older patients (Figure 3). Similar results for the incidence of edentulism worldwide have been reported earlier [6]. Still an increasing prevalence of edentulous patients by age has been reported, with a prevalence of 16% for individuals in the population from 40 to 70 years of age in 1973 [8]. The obvious decrease of edentulous patients during the last 40 years resulting in prevalence of 0.3% (2013) [8] can be interpreted as a result of a higher mortality of edentulous patients in higher age groups than inclusion of new edentulous patients in the edentulous population. This pattern could be associated with various reasons and needs further investigations.

Comparing different age groups of edentulous patients with a reference group from the Swedish population, adjusted for age and gender, the findings from the present study demonstrate that younger edentulous patients (≤59 years of age) have a higher mortality over 10 years of follow-up as compared to their peers in the reference population (*p* < 0.001; Figure 2). The effect of age in relation to all-cause and cause-specific mortality has been reviewed by Koka and Gupta [16]. In contrast to the outcome of the present study, they reported that most of the included studies had shown a stronger association between degree of tooth loss and mortality in the older age group. However, the findings in the present study are supported by findings in the same review indicating a higher mortality for younger edentulous patients than for reference populations of the same age [14]. Thus, the present edentulous population treated with implant-supported dental prostheses between 2009 and 2013 present a comparable mortality pattern with regard to age at surgery (Figure 2) as previously presented in another edentulous implant group, treated between 1986 and 1997 [14].

Another important observation from the present study is that patients treated with dental implants have significantly lower risk for mortality over the 10 years of follow-up time, independent of age at inclusion to the study (Table 2; Figure 2) and regardless of variables related to SES. The effect of replacing missing teeth to prolong life by improving masticatory function and QoL has been debated. In a recent study, denture use was associated with a decreased mortality risk (HR 0.81; CI 0.77 to 0.84) in an elderly population [34]. It was also suggested that the finding was a result of improved masticatory function, better nutritional status, preventing of foreign body asphyxiation, and enhanced QoL [34]. However, some edentulous patients might have been in such a poor condition, where treatment was precluded and adaptation to dentures was regarded as unrealistic. In addition, some studies assume that edentulous individuals who are receiving and accommodating well to dentures or ask for implant-supported treatment alternatives might be healthier and more motivated compared to those choosing only complete denture treatment, or cannot use their dentures [14, 18]. However, the effect of the treatment choice itself is still unclear. It could be speculated that the observation from the present study, with a decreasing mortality risk for the IG compared to the DG, could be a result of healthier and more motivated patients, with better masticatory function, increased patient satisfaction, and higher QoL. The latter suggestion is supported by the findings in the study by Boven et al. [35] who concluded that implant-supported dentures improved the chewing ability and clearly had a positive effect on QoL.

The 10-year mortality risk is significantly higher for the included edentulous patients born in Sweden compared to them who were born abroad and immigrate to Sweden. This is in line with the results from a demographic report from 2016 [36] but in contradiction to results from a Swedish population study from 2005 [37] where the group born in Sweden has significantly lower mortality compared to foreign-born individuals. However, these observations are not based on only edentulous patients, and the prevalence of edentulism and cause of total tooth loss could differ between persons born in Sweden and abroad. It could be estimated that the annual incidence of total tooth loss is about 0.018% and 0.045% in the present two groups, indicating that fewer individuals born in Sweden become edentulous. Complete tooth loss is a result of many factors, but it could be suggested that a higher proportion of persons born in Sweden may lose teeth due to an inflammation-driven, treatment-resistant, periodontitis, which has been reported to be associated with an increased risk of mortality [38].

In general, individuals with higher SES living in urban areas usually have better access to advanced dental care, can afford more costly treatments, and present better oral health [39]. All-cause mortality with regard to degree of tooth loss and edentulism and associated with variables related to SES factors is diffuse and often difficult to interpret [16, 18]. In the present study of edentulous individuals, some variables have been covered and analyzed (Figure 4). However, no significant differences in mortality were observed between the two study groups, related to education, income, and regional typology. Further investigations for better understanding are indicated.

## 5. Limitations

Several limitations can be discussed in the present study. One such limitation is that edentulous patients treated within the national guidelines for dental care funded by the Swedish counties are not included. In 2018, the National Board of Health and Welfare published a report [40] and estimated 0.02% of the Swedish population receive dental care treatment through this funding. However, since only 0.3% of the 40–70 years individuals are edentulous in Sweden [8], it is considered that the number of patients from this specific group is small and should not affect the results in the present study. Another major limitation is the lack of data about cause of tooth loss, the medical history, drug-use, smoking, and other lifestyle factors for the included patients. The choice of implant-supported prostheses or removable dentures may be related to general health factors in the elderly group where healthier and more motivated patients may choose implant-supported prostheses more often. However, younger edentulous patients are fewer in numbers, and they have been shown to present earlier a higher risk for implant loss and early mortality [14, 41, 42]. This may reduce the difference in risk of mortality between the IG and the DG. Still, there are many edentulous patients that chose removable dentures and economy cannot be disregarded. Besides the risk of tooth loss, smoking is a risk factor for several diseases like cancer, cardiovascular diseases, diabetes, and chronic obstructive pulmonary disease and one of the most important risk factors for mortality in the world [1]. However, daily smoking has decreased in Sweden in the last decades, and in the year 2018, only 7% of the population between 16 and 84 years of age were smokers [43]. The number of people that are edentulous smokers today is unknown. The lack of data about smoking in the present population should not have a major influence to the results. The low proportion of edentulous patients in this study may limit the external validity of the present results; other regions with a higher incidence of edentulism may present different results. Thus, this study exemplifies a situation where very few patients become edentulous in the population, only covering the most compromised edentulous patients. Previous data suggests another pattern when the edentulous population includes a larger part of the population who were treated during an earlier period of time [14].

In a previous publication, young edentulous patients with implant-supported prostheses have been shown to have an association between increased mortality and cardiovascular diseases [41]. This earlier study supports the present results, and the cause of death for the patients in the two study groups of the present study would be of interest to further investigate in the future.

## 6. Conclusion

Within the limitations of the present study, the following conclusions were made:

- Completely edentulous individuals show higher mortality compared to the reference population in Sweden matched after age and gender.
- Edentulous patients treated with implant-supported prostheses present statistically significant lower 10-year mortality compared to patients treated with conventional removable dentures in all age groups, independently of gender, SES (education level, equalized disposable income, and regional typology), and country of birth.
- With regard to age, young edentulous patients (≤59 years) demonstrate a higher mortality than the reference population while old implant patients (≥80 years) present a lower mortality.
- The annual incidence of edentulism was estimated to 0.023% in the Swedish population during the inclusion period (2009–2013).
- A higher incidence of edentulism was observed for patients born abroad; however this group showed a lower mortality than patients born in Sweden.
- Low socioeconomic status (SES) was associated with higher mortality in both study groups.

## Figures and Tables

**Table 1:** Multivariable logistic analysis of edentulous patients treated with implants (IG = 2.192 patients) or not (DG = 6.271 patients).

	IG (*N* = 2.192)			DG (*N* = 6.271)		
	OR	95% CI	*p* value	OR	95% CI	*p* value
*Age*						
≤59 years	1.0		<0.0001	1.0		<0.0001
60–79 years	4.0	3.3–5.3		3.4	2.9–4.0	
≥80 years	16.3	10.1–26.2		16.0	13.0–19.7	

*Gender*						
Male	1.0		<0.0001	1.0		<0.0001
Female	0.6	0.5–0.8		0.7	0.6–0.8	

*Education level*						
Tertiary	Excluded after univariable analyses					
Secondary						
Primary						

*Equalized disposable income (EDI)*						
High	1.0		<0.0001	1.0		<0.0001
Intermediate	1.9	1.6–2.3		1.6	1.3–1.8	
Low	3.6	2.6–5.3		4.4	3.6–5.3	

*Regional typology*						
Predominately rural	Excluded after univariable analyses		1.0			<0.05
Intermediate			1.2		1.0–1.3	
Predominately urban			1.3		1.0–1.5	

*Country of birth*						
Abroad	1.0	1.1–2.0	0.004	1.0	1.5–2.0	<0.0001
Sweden	1.5			1.8		

Event estimated “deceased.” Odds ratios and ± 95% confidence intervals are presented as well as *p* values for likelihood Chi^2^ statistics. For the reference level, the OR = 1. *p* values are given for variables within the study groups.

**Table 2:** Multivariable logistic analysis of edentulous patients stratified on age.

	Age-group ≤59 years (*N* = 2709)			Age-group 60–79 years (*N* = 4584)			Age-group ≥ 80 years (*N* = 1170)		
	OR	95% CI	*p* value	OR	95% CI	*p* value	OR	95% CI	*p* value
*Implant*									
Yes	1.0		0.0025	1.0		<.0001	1.0		<0.01
No	1.7	1.2–2.4		1.7	1.4–2.0		1.8	1.2–2.6	

*Gender*									
Male	1.0		0.05	1.0		<.0001	1.0		<.0001
Female	0.8	0.6–1.0		0.7	0.6–0.8		0.5	0.4–0.7	

*Education level*									
Tertiary	Excluded after univariable analyses								
Secondary									
Primary									

*Equalized disposable income (EDI)*									
High	1.0		<.0001	1.0		<.0001	1.0		<.0001
Intermediate	2.0	1.3–2.9		1.7	1.4–2.1		1.0	0.6–1.6	
Low	4.9	3.4–7.1		4.5	3.6–5.5		3.1	1.9–5.2	

*Regional typology*									
Predominately rural	Excluded after univariable analyses								
Intermediate									
Predominately urban									

*Country of birth*									
Abroad	1.0		<.0001	1.0		<.0001	1.0		<0.01
Sweden	1.9	1.4–2.4		1.5	1.3–1.8		1.6	1.1–2.3	

Event estimated “deceased.” Odds ratios and 95% confidence intervals are presented as well as *p* values for likelihood Chi2 statistics. For the reference level, the OR = 1. *P* values are given for variables within the age groups.

## Data Availability

Due to ethical concerns, supporting data cannot be made openly available. The causes of death data used to support the findings of this study were supplied by the National Board of Health and Welfare in Sweden under license and therefore cannot be made openly available. Requests for general access to these data should be made to the National Board of Health and Welfare in Sweden (https://www.socialstyrelsen.se/english). Further information about the causes of death data and conditions for access is available at https://www.socialstyrelsen.se/statistics/statisticaldatabase/causeofdeath.
